# Enhancing adherence in trials promoting change in diet and physical activity in individuals with a diagnosis of colorectal adenoma; a systematic review of behavioural intervention approaches

**DOI:** 10.1186/s12885-015-1502-8

**Published:** 2015-07-07

**Authors:** Deborah McCahon, Amanda J. Daley, Janet Jones, Richard Haslop, Arjun Shajpal, Aliki Taylor, Sue Wilson, George Dowswell

**Affiliations:** 1 Primary Care Clinical Sciences, School of Health and Population Sciences, University of Birmingham, Edgbaston, Birmingham, B15 2TT UK; 2 Critical Care and Perioperative Medical Research Group, Queen Mary University of London, Mile End Road, London, E1 4NS UK; 3 School of Medical and Dental Sciences, University of Birmingham, Edgbaston, Birmingham, B15 2TT UK

**Keywords:** Adenomatous polyps, Colorectal Neoplasms, Exercise, Diet, Intervention studies, Patient adherence, Patient compliance, Behaviour, Review

## Abstract

**Background:**

Little is known about colorectal adenoma patients’ ability to adhere to behavioural interventions promoting a change in diet and physical activity. This review aimed to examine health behaviour intervention programmes promoting change in diet and/or physical activity in adenoma patients and characterise interventions to which this patient group are most likely to adhere.

**Methods:**

Searches of eight databases were restricted to English language publications 2000–2014. Reference lists of relevant articles were also reviewed. All randomised controlled trials (RCTs) of diet and physical activity interventions in colorectal adenoma patients were included. Eligibility and quality were assessed and data were extracted by two reviewers. Data extraction comprised type, intensity, provider, mode and location of delivery of the intervention and data to enable calculation of four adherence outcomes. Data were subject to narrative analysis.

**Results:**

Five RCTs with a total of 1932 participants met the inclusion criteria. Adherence to the goals of the intervention ranged from 18 to 86 % for diet and 13 to 47 % for physical activity. Diet interventions achieving ≥ 50 % adherence to the goals of the intervention were clinic based, grounded in cognitive theory, delivered one to one and encouraged social support.

**Conclusions:**

The findings of this review indicate that behavioural interventions can encourage colorectal adenoma patients to improve their diet. This review was not however able to clearly characterise effective interventions promoting increased physical activity in this patient group. Further research is required to establish effective interventions to promote adherence to physical activity in this population.

## Background

Colorectal cancer is the third most common cancer in the UK, the second most common cause of cancer death and its incidence [1] is increasing. Most colorectal cancers arise from polyps or adenomas, and high-risk adenomas (HRA) are the most likely to become cancerous [2]. One of the aims of the National Health Service Bowel Cancer Screening Programme (NHSBCSP) is to detect and remove colorectal adenomas and thus improve survival [3]. Whilst adenoma removal reduces the risk of colorectal cancer, the underlying risk factors that influence recurrence of ademona remain and the recurrence rate for adenoma has been shown to be relatively high at around 40 % after three years [4].

There is consistent evidence from observational studies that high (>500 g per week) dietary red and processed meat intake and low levels of physical activity cause colorectal cancer [5]. These risk factors are potentially modifiable and behavioural interventions which encourage change in diet and physical activity may reduce risk of recurrence of colorectal adenoma and development of colorectal cancer [6–9].

Through the introduction of the National Health Service Bowel Cancer Screening Programme the rates of detection of adenomas is likley to increase. As such identification of effective interventions to change behaviour associated with risk of colorectal adenoma in this patient group are becoming increasingly important.

Evidence suggests that interventions for populations at increased risk of disease are more likely to be successful than in healthy populations. Compared with the general population, patients with a previous diagnosis of colorectal adenoma are at increased risk of colorectal cancer. This patient population is different to the general population since they have received screening and surgical intervention to remove adenomatous polyps. As such, findings from trials of health behaviour interventions in the general population are unlikely to be generalisable to this patients group.

Previous systematic reviews of exercise and diet interventions for adults have focussed on different types of cancer, types of intervention and various outcomes [10–25]. Data derived from trials with cancer survivors may not however be applicable to this patient group either because colorectal adenomatous ploys are considered precursors to colorectal cancer.

Inadequate adherence in clinical trials contributes to significantly increased study costs, complicates statistical analysis and threatens study validity [26–28]. Clinical trials of behavioural interventions frequently suffer from low levels of adherence with estimates suggesting that between 25 and 50 % of research participants are not adherent [26]. Broadly, adherence can be defined as the extent to which a trial participant acts in accordance with the instructions or recommendations of the research as specified in the study protocol.

The current literature review was undertaken to examine behavioural intervention programmes and determine adherence in RCTs promoting a reduction in consumption of red meat, elimination of processed meat and increased physical activity in individuals with a diagnosis of colorectal adenoma. The aim was to define diet and physical activity interventions to which colorectal adenoma patients are likely to adhere and to use these in the development of a large prospective RCT to assess whether the interventions are effective in changing health behaviour associated with risk of colorectal adenoma.

To achieve this aim it was necessary to i) identify RCTs of dietary and/or physical activity interventions promoting risk reduction in individuals with a diagnosis of colorectal adenoma, ii) summarise data related to protocol adherence and follow-up in these RCTs and iii) characterise the behavioural interventions or elements of these interventions which achieved and sustained maximum adherence.

## Review

### Search methods to identify relevant studies

An electronic search of eight databases (Pubmed, Cochrane, Medline, Embase, PsychINFO, HMIC, Cinahl and BNI) was conducted to capture relevant publications (searches last conducted October 2012). Detailed search strategies were developed for each database (Table 1). Searches were limited to studies involving humans, in English language and published since 2000. Significant advancement in health behaviour research and technology has been made over recent years. This time frame was chosen to enable identification of trials of health behaviour interventions which are most applicable and relevant to a contemporary cohort of patients with colorectal adenoma. All retrieved articles were reviewed to identify additional, relevant RCTs. To ensure consistency in selection, the titles and abstracts of all papers retrieved via the searches were reviewed independently by two reviewers. Papers that did not fulfil the selection criteria were excluded. Full papers were obtained for the remaining studies and two reviewers read and independently applied the selection criteria. The two reviewers met to resolve any disagreement and reach consensus.

**Table 1:** Search terms

Physical activity	Diet	Diet (Cont)	Compliance	Medical
Exercis*	Diet*	Venison	Adherence	Cancer
Exercise test	Diet restriction	Veal	Attitude to health	Adenoma*
Exercise Tolerance	Diet, protein-restricted	Bacon	Behavio?r change	Colorect*
Exercise therapy	Diet, fat-restricted	Sausages	Health behavio?r*	
Physical endurance	Meat	Ham	Behavio?r modification	
Physical exertion	Meat products	Hotdogs	Lifestyle changes	
Physical fitness	Processed meat	Burgers	Patient* attitude	
Physical activity	Red meat	Meatloaf	Patient* compliance	
Physical training	Beef	Salami	Patient* reported outcomes	
Motor activity	Lamb	Corned beef	Patient* participation	
Movement	Pork	Tinned meat	Patient satisfaction	
Motion therapy	Rabbit		Readiness to change	
	Venison		Refusal to participate	
	Veal			
	Filters:	RCTs		
		Humans		
		English language		

### Selection criteria

#### Inclusion criteria

(i) RCTs with a population of adults with a previous diagnosis of colorectal adenoma without a previous diagnosis of colorectal cancer.
(ii) RCTs which evaluated a behavioural intervention aiming to promote change in physical activity and/ or diet.
(iii) RCTs reporting data related to adherence as either a dichotomous or continuous variable.

Other outcomes of interest were retention, attrition and reasons for drop-out. RCTs were not excluded, however, if data related to these outcomes were not reported. Meta-analysis and systematic reviews were employed as sources of additional RCTs only.

#### Exclusion criteria

(i) RCTs in cancer patients or cancer survivors
(ii) RCTs of prevention in cancer patients
(iii) RCTs in which adherence data could not be extracted.

### Quality assessment

The quality of each included RCT was assessed using the Critical Appraisal Skills Programme RCT checklist [29]. The quality of each included RCT was assessed by two of the reviewers (JJ and RH) with disagreements being resolved by discussion.

### Data extraction

For each of the included RCTs, the paper was read in full by two reviewers (DM and AS). Data were extracted using a proforma specifically designed to record key information related to (i) study design (ii) population characteristics (iii) characteristics of the intervention including: type of intervention; mode, location and delivery of interventions; (iv) type of intervention provider (v) duration, intensity and frequency of the intervention. Data to enable calculation of adherence, frequency and methods of assessment of adherence and reasons for drop out were also extracted.

### Outcomes of interest of this review

There were four main outcomes of interest of this review. Firstly, this review focused upon whether participants received/attended the intervention or its components, as described in the study protocol. Participants needed to have attended or engaged with each of the scheduled components of the intervention to be considered fully adherent in this outcome (intervention adherence). The second outcome of interest was the extent to which participants met the dietary and/or physical activity goals of the intervention. To be classified as adherent for this outcome, participants had to adhere to ≥50 % of the diet and/or physical activity goals of the intervention. In health behaviour, it is difficult to give a precise definition or cut-off for when behaviour is deemed acceptable or not and this may vary from one context or population to another. A judgment on what such a cut-off might be was therefore required. Following much discussion and consideration, a minimum threshold of 50 % was selected because this meant at least half of the sample had achieved at least half of the intervention. This was considered in light of the fact most people in the modern Western world are sedentary and do very little physical activity–so a shift in physical activity from very little to a minimum adherence of 50 % of a physical activity intervention is not insignificant and even small changes in behaviour can be clinically worthwhile [30]. Given that participants who do well in the intervention are more likely to agree to follow-up, the third outcome was the follow up rate in the intervention group to enable comment upon the burden and acceptability of the intervention. A fourth and final outcome of interest was reported reasons for drop out.

### Methods of synthesis

Since the focus of this review was identification and characterisation of behavioural interventions that maximise adherence in RCTs promoting behavioural change in adenoma patients, it was not appropriate to conduct a statistical analysis. Data were therefore subject to a narrative synthesis.

### Results of the search

**Fig. 1:**
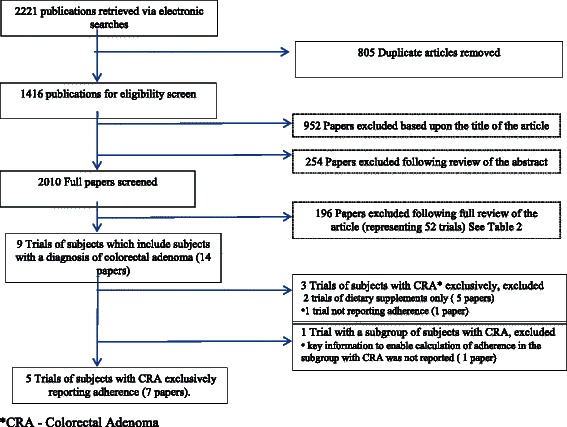
Results of the search strategy

Figure 1 shows the outcome of the search process and application of the selection criteria. The electronic searches identified 2221 potentially relevant articles. Following removal of 805 duplicates, 1416 papers remained. A further 1206 of these articles were excluded following review of the title or abstract and 196 articles were excluded after a full review of the article. The reasons for exclusion are provided in Table 2. The 14 remaining articles reported on nine RCTs which included individuals with a diagnosis of colorectal adenoma. Two of these RCTs were excluded from further review because they reported on RCTs of a dietary supplement and two RCTs were excluded because calculation of adherence was not possible. Five RCTs of a diet and/or physical activity intervention in colorectal adenoma patients were included in the current review [31–35].

**Table 2:** Reason for exclusion of papers

Reason for exclusion	n (%)
Trials in breast cancer patients or survivors	66 (34)
Non RCT (includes systematic reviews)	53 (27)
Prevention trials/ trials in healthy subjects	34 (17)
Trials in prostate cancer patients or survivors	11 (6)
Trials in subjects with breast or prostate cancer	6 (3)
Trials in subjects with colorectal cancer	6 (3)
Trials in other cancer patients or survivors	20 (10)
Total	196

### Description of included trials

The characteristics of the five RCTs included are summarised in Tables 3, 4 and 5. The Minnesota Cancer Prevention Research Unit (Minnesota CPRU) [31] trial and the Polyp Prevention Trial (PP trial) [32, 36] evaluated the impact of a behavioural intervention upon diet alone and the Bowel Health for Better Health (BHBH) [34], PREVENT [33] and the BeWEL [35] trials examined the impact of a behavioural intervention upon diet and physical activity (Tables 3, 4 and 5). In total, 1932 adenoma patients were randomised to receive these behavioural interventions. The majority of trial participants were aged 40 years or more, Caucasian and had received at least 15 years of education. All five publications reported that the behavioural interventions were successful in achieving change in diet and/or physical activity in adenoma patients (Table 3).

**Table 3:** Characteristics of included trials

Author, pub date and location	Trial name and acronym Eligibility criteria	Type of intervention	Trial duration and number of participants recruited	Run in phase	ITT analysis	Characteristics of participants	Summary of trial findings as reported in publication
Smith Warner 2000 ^31^	Minnesota cancer prevention research unit diet intervention trial–Minnesota CPRU	Diet	12 months *n* = 100	No	Yes	Mean age 59 years	Individuals at high risk for development of colorectal cancer can successfully increase F&V intake and maintain that increase over a year period.
USA	30-74 years with a diagnosis of colorectal polyps in preceding 5 years, no medical conditions or chronic disease.					71 % male, 99 % Caucasian, mean number of years in education was 15	
Lanza 2001 ^32^	Polyp Prevention Trial–PP trial	Diet	4 years *n* = 1037	Yes, 4 day food record and frequency survey	Yes	Mean age 61 years	Free-living individuals can alter their eating patterns in a significant way given appropriate support
USA	≥35 years having removal of ≥ 1 colorectal adenomas removed within past 6 months, no history of colorectal cancer					66 % male, 12 % minority race, 65 % higher than high school education	
Emmons 2005 ^33^	Project PREVENT	Diet and physical activity	8 months *n* = 591	No	Yes	46 % aged 40–59 years and 54 % aged over 60 years	PREVENT was effective in helping adenoma patients to change and reduce behavioral risk factors and behavioral change is possible in this population
USA	40-65 years with a adenomatous colon polyp removed within 4 weeks of recruitment, no history of colorectal cancer					56 % male,83 % white, non Hispanic, 74 % higher than high school education	
Caswell 2009 ^34^	Bowel Health to Better Health–BHBH	Diet and physical activity	12 weeks *n* = 41	No	Not explicit	Mean age 62 years	Population is responsive to minimal contact intervention to promote positive change in diet
UK	50-74 years					71 % male, 100 % Caucasian	
	≥1 colorectal adenoma, no evidence of colorectal carcinoma or metaplastic or hyperplastic non-adenomatous polyps					Index of multiple deprivation low 20 %, medium 40 %, high 40 %	
Anderson 2014 UK ^35^	BeWEL, 50–74 years, undergone polypectomy for adenoma, able to undertake physical activity	Diet and physical activity	12 months *n* = 163	No	Yes	Mean age 63.5 years, 74 % male, 100 % white, 86 % equal to higher than secondary school education	Significant weight loss can be achieved by a diet and physical activity intervention initiated within a national colorectal cancer screening programme

**Table 4:** Characteristics of the intervention

	Frequency, duration and intensity of intervention	Behavioural components of the intervention	Educational complements of the intervention	Affective components of the intervention	Mode and intensity of delivery of the intervention (including total number of hours of delivery)
Smith Warner 2000 ^31^	^a^ Increase fruit and vegetable intake to at least 5–8 servings per day	Nutrition counselling; goal setting, verbal commitments to behavioural intentions, skill development, planning and self monitoring. Memory aids; Fridge magnets, visit reminder cards and birthday cards.	Written educational materials; tip sheets, a cookbook and quarterly newsletters	Frequent intervention visits with nutritionist. Spousal support encouraged.	Clinic based, individual sessions provided by nutritionist at baseline, month 1, 4, 7 and 10.
		Positive reinforcement and feedback			Insufficient data provided to enable calculation of the total number of hours counselling provided as part of the intervention
Lanza 2001 ^32^	Increase; daily fruit and vegetable consumption to 5–8 servings per day	Individual counselling sessions to set personal goals, promote behaviour modification, motivate, skill building, and self monitoring	Provision of standardised education materials on nutrition and behavioural modification	Frequent group counselling sessions and telephone contact 6 monthly to resolve difficulties and discuss progress	Clinic based individual and group sessions, weekly counselling for 6 weeks, biweekly for 6 weeks, monthly sessions thereafter. Year 2, 3&4 monthly group sessions provided by a dietician.
	daily fibre to 4.30 g fibre/mJ per day and consume 20 % less energy from fat		Annual education campaigns (1 for each diet goals)		50 h of counselling in total
Emmons 2005 ^33^	150 min per week, moderate intensity physical activity	Motivational and goal setting initial counselling telephone call.	Provision of a personal profile detailing risk status and highlighting the importance of risk factor reduction. Written materials; tip sheet, guide book, fitness brochure and Q&A sheet	Help to develop coping skills, confidence and self efficacy.	Home based individual initial counselling telephone call followed by four calls at monthly intervals and four mail shots provided by a health educator.
	Increase daily fruit and vegetables to ≥5 servings and weekly red meat to ≤3servings, increase vitamin and reduce alcohol intake and stop smoking	Skill building; planning and self monitoring			6.5 h of counselling in total
		Printed progress reports with positive reinforcement and feedback			
		Tailored self help materials			
Caswell 2009 ^34^	30 min physical activity per day, moderate	Individual counselling assessment and goal setting session, personalised programme explained,	General cancer prevention literature, physical activity literature and fruit and vegetable literature including recipes	Motivational letters with specific tailored guidance based upon self efficacy and ability. Social support identified	Clinic based, individual 2 h session followed by 3 personalised mail shots, ad hoc telephone support provided by researchers. 2 h counselling in total
	^a^ Consume ≥5 serving of fruit and vegetable per day and increased daily fibre intake	Action planning and self monitoring encouraged			
Anderson 2014 ^35^	Target goal was 7 % reduction in body weight,	Individual counseling with motivational interviewing, goal setting, positive reinforcement and feedback, self monitoring. Personalised energy prescription and tool kits provided (shopping bag, water bottles with study logo, body weight scales, physical activity equipment (hand weights, DVDs)	Provision of the British Heart foundation booklet ‘so you want to lose weight for good’	Support from spouse/ friend encouraged. Motivational interviews exploring self assessed confidence and personal values concerning weight. Telephone contact offered to discuss and overcome relapse	During the first 3 months trained lifestyle counsellors provided 3 x 1 h, individual face to face sessions. Sessions where home and/or clinic based. Followed by 9 monthly 15 min telephone calls. Total number of hours contact 5.25 h over 12 months
	150 min per week, moderate intensity physical activity				
	Increase daily fruit and vegetable consumption to 5 portions per day,				

^a^ Intervention is effective for promoting behavioural change in adenoma patients based upon ≤50 adherence to the behavioural goals of the intervention

**Table 5:** Adherence outcomes

Author name and pub date	Intervention adherence	Adherence to the behavioural goals of the intervention	Follow-up rate	Reasons for withdraw from the intervention	Method and frequency of assessment of adherence
Smith Warner 2000 ^31^	Based upon clinic attendance, Attendance averaged 93 % of all clinic visits	^a^ 86 % met or exceeded the fruit and vegetables goals of the intervention	88 %	2 % (2/100) inappropriately randomised, 10 % (10/100) reason not reported	Baseline and at 3, 6, 9 and 12 months.
					Objective and subjective; diet records and measurement of biological markers (concentrations of carotenoids, lipids, sodium and potassium).
					Attendance monitored by intervention provider
Lanza 2001 ^32^	Not specified and inadequate data reported	Dietary goals met;	89 %	4 % (43/1037) died,	Baseline and end of each year plus unannounced 24 h dietary recall in 10 % of participants each year.
Supplementary adherence data was extracted from Sansbury 2009 ^36^		25.6 % (210/821) met 9–12 goals		7 % (71/1037) withdrew due to illness, moved clinical centre, did not wish to continue	Subjective and objective, food frequency questionnaire, 4 day food records and 24 h dietary recalls and measurement of biological markers (concentrations of carotenoids and lipids)
		45 % (366/821) met 4–8 goals			
		29.8 % (245/821) met 0–3 goals.			
		Data reported did not allow distinction between the 3 dietary goals being evaluated			
Emmons 2005 ^33^	60 % received 4 to 5 intervention telephone calls conducted by health educators	Physical activity goals met by 13 % (76/591)	83 %	No dropout reported	Baseline and end of 8 month study period. Subjective only–22 item food frequency and 24 item (CHAMPS) activity questionnaire.
		Dietary targets met;			Receipt of telephone calls monitored by intervention provider
		20 % (118/591) met fruit and vegetable goals			
		18 % (104/591) met red meat goals			
Caswell 2009 ^34^	Insufficient data reported to enable calculation	Physical activity goals met by 47 % (15/32)	78 %	Dropout calculated as 22 % (9/41)	Baseline and end of 12 week study period. Subjective only–24 h recall of fruit and vegetables and food frequency questionnaire to provide fibre consumption score (recorded mid week) and 7 day physical activity recall questionnaire.
		Dietary targets;			
		^a^ Fruit and vegetable goals met by 84 % (27/32)			
		^a^ Fibre goals met by 53 % (17/32)			
Anderson 2014 ^35^	97 % attended all face to face sessions (3 sessions)	Data reported do not allow calculation of the % achieving 150 min per week, moderate intensity physical activity	91 %	15 participants withdrew, 7 gave no reason,	Baseline, 3 and 12 months.
	59 % completed all of the 9 planned telephone calls	Dietary targets;		3 withdrew due to health concerns, 1 moved, 2 reported personal reasons and 2 were unable to commit.	Subjective and objective, self reported daily diary and food frequency questionnaire measurement body weight, waist circumference, blood pressure, and of biological markers (e.g., total, low and high density lipoprotein cholesterol, triglycerides, glucose, glycated haemoglobin and insulin)
	95 % completed 5 of 9 telephone calls	^a^ Fruit and vegetable goals met by 73 % met.			SenseWear armband worn for 7 days to measure daily expenditure and minutes of moderate intensity exercise.
					Trained lifestyle counsellor recorded attendance

^a^ Intervention is effective for promoting behavioural change in adenoma patients based upon ≤50 adherence to the behavioural goals of the intervention

### Characteristic of the behavioural intervention

In all five RCTs, participants were asked to meet or exceed current diet and/or physical activity recommendations for risk reduction at the general population level (Table 4).

The intervention in each of the five RCTs comprised a combination of behavioural, educational and affective approaches to promote behavioural change. Behavioural components of the intervention were based upon cognitive behavioural psychology and employed techniques such as negotiation and goal setting and encouraged planning, self monitoring and skill building. In addition, the Minnesota CPRU, PREVENT and BeWEL trials provided positive reinforcement and feedback. The Minnesota CPRU trial also used fridge magnets and birthday cards as memory aids to maintain motivation and adherence. Tool kits of items such as pedometers and shopping bags and water bottles with trial logos were provided to participants of the BeWEL trial. Other equipment such as weighing scale, kitchen gadgets, physical activity equipment (e.g., exercise DVDs, hand weights and hoola hoops) were available, on loan also.

The educational materials delivered as part of the diet intervention generally provided information on nutrition and advice on ways to modify lifestyle to concur with target recommendations of the intervention. To highlight the importance of risk factor reduction, the PREVENT intervention provided information on personalised risk profiles in addition to distribution of general literature related to cancer prevention. Affective components of the intervention focused upon development of coping skills, confidence and self efficacy and provision of emotional support. In the Minnesota CPRU, BHBH and BeWEL trials support from a friend or partner was encouraged. Diet interventions were delivered by dedicated dieticians and/or nutritionists. Trained lifestyle counsellors delivered the diet and physical activity intervention in the BeWEL trial. No exercise experts were involved with development and/or delivery of the physical activity interventions. The interventions were delivered at individual counselling session in the Minnesota CPRU, PP, BHBH and BeWEL trials. The PREVENT trial employed a combination of individual and group sessions.

### Intervention adherence

Intervention adherence was reported in the Minnesota CPRU, PREVENT and BeWEL trials only. Full intervention adherence was not, however, achieved in either of these trials. In the Minnesota CPRU trial, 93 % intervention adherence was reported based upon attendance at all four intervention visits. The PREVENT trial reported that 60 % of participants received four of the five counselling telephone calls. The BeWEL trial reported that 97 % attended all the face to face sessions (3 sessions) and 59 % completed all of the 9 planned telephone calls (Table 5).

### Adherence to the behavioural goals of the intervention

Across the five RCTs, adherence to the dietary goals of the intervention ranged from 18 to 86 % and adherence to the physical activity goals of the intervention ranged from 13 to 47 % in the RCTs encouraging increased physical activity (Table 5).

In terms of effectiveness, the Minnesota CPRU, BHBH and BeWEL interventions were successful in achieving ≥ 50 % adherence to the behavioural goals of the intervention. In the Minnesota CPRU, diet only interventions achieved 86 % adherence to the fruit and vegetable goals of the intervention. The BHBH intervention, which promoted change in both diet and physical activity, was more effective with respect to diet, achieving 84 % adherence to the fruit and vegetable goals, 53 % adherence to the fibre goals and only 47 % adherence to the physical activity goals of the intervention. The BeWEL diet intervention achieved 73 % adherence to the fruit and vegetable goals. The PREVENT intervention, which promoted change in both diet and physical activity, was ineffective and failed to achieve adherence of ≥50 % with respect to any of the behavioural goals of the intervention. The effectiveness of the PP intervention could not be defined because adherence was assessed at multiple points and divided into three subgroups based upon total number of goals met during the trial period (Table 5).

### Follow-up rate

Follow-up rate was generally high, ranging from 78 to 89 % in the RCTs of promoting change in diet and 78 %-91 % in RCTs encouraging change in both diet and physical activity. The reasons for withdraw or loss to follow-up were reported in the Minnesota CPRU, BeWEL and PP trials only. The Minnesota trial reported that 2 % of participants were inappropriately randomized and a further 10 % withdrew or were lost to follow-up. In the PP trial, 4 % were lost to follow-up. In the BeWEL trial, 9 % withdrew (Table 5).

### Reasons for drop out

Only the BeWEL and PP trials reported reasons for drop out. 7 % of the PP trial participants discontinuing due to illness, no longer wishing to participate or moving to a health centre not participating in the trial (Table 5).

### Methodological quality of the included trials

A meta analysis of trial data was not possible due to the heterogeneity in trial design and outcomes reported. Data related to trial quality was therefore subject to narrative synthesis. Trial quality was assessed using the Critical Appraisal Skills Programme RCT checklist and all trials were considered to be of high quality (scores ranging from 7.5 to 9 out of 10). The lack of reporting of research personnel blinding and reasons for participant withdraw from the study were the most commonly recorded methodological weaknesses. Two of the RCTs also failed to provide details of the required sample size and/or to comment upon whether the study was adequately powered to detect a significant difference between the two study arms [31, 32].

## Discussion

### Summary of main findings

This review identified two behavioural interventions that were effective in achieving ≥50 % adherence to a diet intervention and encouraging change in fruit and vegetable intake in colorectal adenoma patients. The effective diet interventions were grounded in social cognitive theory with the initial intervention counselling session being delivered individually during a clinic based consultation. These interventions also encouraged participants to identify social support and provided personalised, printed educational materials and recipes to aid behavioural change.

However, the physical activity interventions reviewed did not achieve similar levels of adherence to the goals of the intervention and as such, were ineffective for promoting increased physical activity in colorectal adenoma patients. Inaccuracies in self reporting of adherence due to recall bias and discrepancies between researcher and participant definition of moderate intensity activity may explain low adherence rates to these physical activity interventions. Of additional note is the lack of involvement of an exercise specialist in development and/or delivery of these physical activity interventions.

Intervention adherence could not be compared across all five RCTs due to either lack of reporting or heterogeneity in reporting. Furthermore, full intervention adherence was not achieved in any of the RCTs reviewed.

Of particular note, the follow-up rate in all five trials was high which may indicate that some aspects of the interventions used in these trials are acceptable to this patient group. However since the trials all employed complex interventions and do not report adherence to individual components of the intervention it is difficult to identify which of the components were more acceptable than others.

Overall, data relating to intervention adherence and reasons for drop out provided little insight with respect to the characteristics of the interventions to which this patient group are most likely to adhere. The five RCTs reviewed were relatively homogeneous with respect to the demographics of the populations studied and the nature, content and target recommendations of the behavioural interventions. However, these RCTs were heterogeneous regarding the timing of the intervention in relation to the diagnosis of colorectal adenoma, the duration of the RCT and the intensity of delivery of the intervention. Overall, the methodological quality of the included RCTs was good.

The physical activity and diet interventions were very similar with respect to the behavioural, educational and affective strategies employed to promote behavioural change. Furthermore, these strategies have been shown to be effective in increasing adherence to physical activity in cancer patients and survivors [37, 38]. The reasons why the physical activity interventions were less effective in this population are therefore unclear. It is possible that colorectal adenoma patients perceive change in diet to be more easily achievable than change in physical activity, and as such, require greater motivation, self efficacy and confidence to adhere to target recommendations for physical activity. A more structured, maximum contact, patient focused, physical activity programme than that provided by the PREVENT and BHBH interventions may therefore be necessary to promote adherence in this patient group.

There is evidence to suggest that colorectal adenoma patients are largely unaware of the implications of their diagnosis and may not view themselves as being at increased risk of colorectal cancer [39–42]. Since people are more amenable to behaviour change following a health event or scare [43], it is possible that improved communication of personal risk with respect to recurrence of colorectal adenoma and progression to colorectal cancer at the time of colorectal adenoma diagnosis will enhance adherence to behavioural interventions in this population. Personalized risk information was provided in one of the behavioural interventions; unfortunately this intervention was ineffective with regard to promoting adherence or change in this population. However this intervention did target five other risk factors for colorectal adenoma recurrence in addition to encouraging increased physical activity. This provides another possible reason why it was ineffective for promoting change in physical activity in colorectal adenoma patients. Interventions promoting change in multiple risk factors are inherently more complex to deliver and assess and results are more difficult to interpret. From the patient perspective, change in multiple behaviours is also much more challenging and additional barriers and facilitators to change need to be considered when designing behavioural interventions to promote change in multiple risk factors.

### Strengths and limitations

This is the first systematic review to examine behavioural intervention programmes and determine adherence to interventions which promote change in diet and/or physical activity in adenoma patients. As such, this review has provided a useful insight into the ability of adenoma patients to adhere to diet and physical activity interventions. Further research is however required to identify physical activity interventions to which colorectal adenoma patients are likely to adhere. Only three of the five RCTs eligible for inclusion in this review examined the impact of a behavioural intervention upon physical activity making it difficult to draw any meaningful conclusions. Moreover, only one RCT promoting a reduction in red meat consumption was identified meaning the aims of this review were not fully met. However, this review did capture all RCTs of diet and/or physical activity in colorectal adenoma patients published in the last 14 years. There is a risk of publication bias because unpublished RCTs were not included. Similarly, limiting literature searches to English language publications may impact upon language bias. However, the effect of this is likely to be small as it is unlikely many if any studies were missed that would have been included in this review.

## Conclusion

This review identified two interventions which were effective in colorectal adenoma patients for promoting change in diet and successfully achieved at least 50 % adherence to the goals of the diet intervention. However, this review failed to identify effective interventions for promoting adherence to physical activity in this patient group. Derivation of a physical activity intervention to which colorectal adenoma patients are likely to adhere was, therefore, not possible. Future research should focus upon interventions promoting change in physical activity alone and which involve an exercise specialist in the design and delivery of the intervention. Provision of personalized risk information should also be considered to promote adherence to physical activity interventions in this patient group.

## Abbreviations

BHBH: Bowel Health for Better Health
CPRU: Cancer Prevention Research Unit
HRA: High Risk Adenoma
NIHR RfPB: National Institute for Health Research, Research for Patient Benefit
NHSBCSP: National Health Service Bowel Cancer Screening Programme
PP: Polyp Prevention
RCT: Randomised Controlled Trial.
